# Flow cytometry analysis of the microbiota associated with the midguts of vector mosquitoes

**DOI:** 10.1186/s13071-016-1438-0

**Published:** 2016-03-22

**Authors:** Tibebu Habtewold, Luc Duchateau, George K. Christophides

**Affiliations:** Department of Life Sciences, Imperial College London, London, UK; Department of Comparative Physiology and Biometrics, University of Ghent, Ghent, Belgium

**Keywords:** *Anopheles Coluzzii*, Microbiota, Midgut homogenate, Flow cytometry, Propidium Iodide, Live, Dead, discrimination, Fixed cells

## Abstract

**Background:**

The scientific interest to understand the function and structure of the microbiota associated with the midgut of mosquito disease vectors is increasing. The advancement of such a knowledge has encountered challenges and limitations associated with conventional culture-based and PCR techniques.

**Methods:**

Flow cytometry (FCM) combined with various cell marking dyes have been successfully applied in the field of ecological microbiology to circumvent the above shortcomings. Here, we describe FCM technique coupled with live/dead differential staining dyes SYBR Green I (SGI) and Propidium Iodide (PI) to quantify and study other essential characteristics of the mosquito gut microbiota.

**Results:**

A clear discrimination between cells and debris, as well as between live and dead cells was achieved when the midgut homogenate was subjected to staining with 5 × 103 dilution of the SGI and 30 μM concentration of the PI. Reproducibly, FCM event collections produced discrete populations including non-fluorescent cells, SYBR positive cells, PI fluorescing cells and cells that fluoresce both in SYBR and PI, all these cell populations representing, respectively, background noise, live bacterial, dead cells and inactive cells with partial permeability to PI. The FCM produced a strong linear relationship between cell counts and their corresponding dilution factors (*R*^2^ = 0.987), and the technique has a better precision compared to qRT-PCR. The FCM count of the microbiota reached a peak load at 18 h post-feeding and started declining at 24 h. The present FCM technique also successfully applied to quantify bacterial cells in fixed midgut samples that were homogenized in 4 % PFA.

**Conclusion:**

The FCM technique described here offers enormous potential and possibilities of integration with advanced molecular biochemical techniques for the study of the microbiota community in disease vector mosquitoes.

## Background

Mosquito vectors of human pathogens house a diverse population of commensal microbiota in their midguts. The abundance and composition of midgut microbiota communities change dramatically after a blood meal [1, 2]. Such changes are influenced by microbial intra-and inter-species interactions, mosquito immune responses, nutrient availability and the pH of the midgut. In turn, the midgut microbiota influence the vectorial efficiency of mosquitoes both by interacting directly with the pathogens that are ingested with the blood meal or indirectly by triggering the immune response of the mosquito [3–6]. As the midgut microbiota are potential targets for disease control, the study of their interactions with the mosquito vector and pathogens has lately received great attention [6–8]. One aspect of such investigations involves establishing the impact of mosquito immune and immune-related genes on the abundance and composition of gut microbiota using *RNAi* based gene-silencing techniques, where homologous mRNA of the target gene is destroyed by the action of dicer machinery [9, 10]. Large changes in the gut microbiota load and compotation occur when receptors of mosquito Immuno-deficiency (Imd) signaling pathway [11], Immunomodulatory peroxidase (IMPer) [12] or other midgut receptor genes, such as including the fibronectin type-III domain proteins (FN3D1-3) [13], are silenced.

Efforts to isolate and characterize the microbiota in the midguts of disease vector mosquitoes date back to the 1960’s [14–16]. Until recently, the conventional culture-based techniques have been used in such studies [2, 17–20]; however, 40–90 % of the gut bacteria are uncultivable or only grow under special conditions and are not observed using culture-dependent techniques [21–23], leading to a non-representative assessment and underestimation of the abundance of the microbiota populations. These limitations have been eliminated with the development of culture-independent techniques. Such techniques are often based on polymerase chain reaction (PCR) of the microbial *16S rRNA* genes [24–26]. The two most common techniques include quantitative real-time PCR (qrtPCR) and microbiome sequencing [6, 27–29]. However, these approaches suffer too from a variety of other limitations including the inability to discriminate between DNA from dead and live bacteria, and between extracellular and intracellular DNA [30, 31].

FCM combined with various cell-staining techniques has been successfully applied in the field of ecological microbiology. Bacterial cells are marked with fluorescent-labelled antibodies, oligonucleotides or general DNA-binding fluorescent dyes such as SYBR green and PI before being subjected to FCM analysis. SYBR can enter both live and dead cells, but the PI is membrane impermeable and it enters only dead cells or cells with compromised membrane. In membrane compromised cells, both SYBR and PI molecules access the nucleic acid and, respectively, bind the DNA minor-groove and intercalate in the DNA. Double staining with the dye pair results in the radiationless fluorescence energy transfer (FRET) from SYBR (donor molecule) to PI (accepter molecule). This leads to a reduction in the SYBR fluorescence intensity and an increase in the PI emission intensity. As a result, the membrane (dead cells) fluoresce only PI. This phenomenon has been exploited in previous studies aimed at discrimination between living and dead bacteria [32, 33].

Here, we applied a FCM-based technique for direct analysis of midgut microbiota in disease vector mosquitoes and discriminate live and dead cells. The FCM technique was evaluated for reliability and precision in measuring the microbiota cells in the gut samples. Also, the efficiency of the technique in quantifying the microbiota in fixed midgut samples was determined.

## Methods

### Ethics statement

The protocol for infecting mice with *P. berghei* and *P. yoelii was* approved and carried out at the Imperial College London under the UK Home Office License PPL70/7185.

#### Mosquito colonies and maintenance

The *Anopheles gambiae* strain N’gousso M-form (a laboratory-strain colonized in 2006 from field mosquitoes collected around Yaoundé, Cameroon), now formally named as *Anopheles coluzzii* [34], was used in these experiments. The mosquitoes were reared and maintained at 27 °C, 70 % relative humidity and 12-h light/dark cycle. Adult mosquitoes were fed on 10 % sucrose cotton pads.

#### Blood feeding

Female mosquitoes 3–5 days old or 3 days post *dsRNA* injection were offered human blood using the membrane feeding system and were maintained on sugar solution until midgut dissection. To ensure aseptic midguts, mosquitoes were given antibiotic mixture of penicillin (10 units/mL)–streptomycin (10 μg/mL) and gentamycin (200 μg/mL) both in sugar solution as well as blood meal.

#### Preparation of midgut homogenate for FCM

Midguts were dissected on sterile glass-slides placed on ice. The midguts were either transferred to Eppendorf® tubes individually or as pools and homogenized by pipetting up and down in ice cold PBS (live homogenate) or 4 % paraformaldehyde (PFA) in PBS (fixed homogenate).

#### Cell culture

LB broth liquid media (20 ml) in 250-mL wide-neck Erlenmeyer flasks was inoculated with midgut homogenate from mosquitoes that obtained a blood meal 24 h earlier. The culture was kept overnight on a shaker at 37 °C. Next morning, the cells were pelleted and re-suspended in fresh culture media and left on the shaker for 4 h to obtain bacterial cells at the exponential growth phase. The cells were equally split into two tubes, pelleted and re-suspended in 1 ml acetone and 1 ml PBS, respectively.

#### Fluorescent staining

Standard nucleic staining protocol combining a cell-permeant fluorochrome SGI (Invitrogen, UK) and the cell-impermeant PI (Invitrogen, UK) was used to stain live homogenate samples, whereas fixed samples were stained with SGI alone. The final staining volume for both live and fixed samples was 350 μl. When enumeration of the microbiota was required, samples were spiked with 25 μl CountBright beads (Life Sciences, UK).

Several concentrations of SGI and PI were tested independently to determine the optimal conditions for the separation between bacterial cells and background, and between live and dead cells. These tests were carried out in pools of 10 midguts from aseptic mosquito; a 10^th^ of the homogenate was used for each FCM analysis. Before adding the SGI and PI, the 2 μl bacterial cells from each group (described above) were spiked to the midgut homogenate samples and incubated for 15 min at room temperature. Finally, the mixture was passed through FCM machine.

The optimal combination of SGI/PI was used to stain live samples in subsequent experiments. Fixed samples were stained with SGI only.

#### FCM analysis

The following FCM machines were used in this study: (i) FACS Calibur flow cytometer (BD Biosciences, USA) was used for an initial proof of concept of direct FCM analysis of midgut microbiota; (ii) BD LSRFortessa™ Cell Analyzer (Becton, Dickinson and Company, BD Biosciences, and San Jose, CA USA) was used to evaluate and optimize the fluorescence signal on SYBR and PI channels; and (iii) BD FACSAria™ III instrument (Becton, Dickinson and Company, BD Biosciences, and San Jose, CA USA) was used for cell sorting.

SGI was excited by a 488 nm (50 mW) laser and collected through a 530/30BP filter with a preceding 502LP filter. PI was excited by a 561 nm (50 mW) laser and the resultant fluorescence collected through a 610/20BP filter with a preceding 600LP filter. The voltages were set on both fluorescent parameters so that the SYBR positive cells and PI positive cells were both on scale and above threshold. All parameters were displayed as height measurements on a logarithmic scale. Dual Boolean ‘OR’ thresholds were established using unstained cells so that a minimal number of events were present at the threshold limits. The compensation was calculated using single stained fixed cells. Tubes containing stained cells were vortexed for 20 s before acquisition on a low flow rate (approximately 12 μL/min), and at least 20,000 events were collected.

First, the forward and side scatter of cells was evaluated in a bivariate dot plot. The population was gated by the relative value of the forward and side scatter to eliminate debris. The gated population was further divided according to SYBR emission (on the abscissa axis) and the PI emission (on the ordinate axis) in a bivariate dot plot. An additional bivariate plot displaying bead fluorescence against time allowed the stability of the acquisition rate to be monitored so that any perturbations that had the potential to affect the count calculations could be identified and excluded. The counting calculations were performed according to manufacturer’s directions.

For cell sorting, the FACSAria III was run at 70 PSI using a 70 μm nozzle and the cells were sorted into tubes containing PBS. Cell sorts were concentrated by spinning down at 500 rpm for 10 min and examined under a fluorescent microscope or plated on LB agar medium.

#### Assay repeatability/intra-assay precision

The bacterial load in midgut samples was determined using three parallel FCM and qrtPCR-based assays, using the same midgut homogenate sample. First, midguts were isolated from 10 mosquitoes that obtained blood meal 24 h earlier, and were homogenized in 1 ml PBS and split into two tubes of 500 μl. Each tube was assigned to one of the assays.

For FCM measurements, the homogenate sample was brought to 1.2 ml volume with PBS/PFA (4 %). A third of the homogenate sample was removed and stained with SYBR dye to enumerate the microbiota cells. This assay was repeated after 3 and 6 days using the remainder of the homogenate sample that was stored at 4 ^o^C. The number of bacteria per microliter of gut homogenate was determined by adding 25 μl of bead suspension (containing 990 beads/μl) before FCM analysis. The total count per sample was calculated as the ratio of the number of events in the bacterial cell population and the number of events in the bead population, multiplied by the ratio of the total number of beads used in the test and the final volume of the test sample. The remaining half of the midgut homogenate was used in qrtPCR assays as described below.

#### qrtPCR analysis

Genomic DNA (gDNA) was extracted from whole midgut homogenate, pellets of the homogenate after spinning or from supernatant only. The bacterial cells in the samples were first lysed with a 40 mg/ml lysozyme solution incubated for 1 h at 37 °C and then subjected to gDNA extraction using the DNeasy Blood & Tissue kit (QIAgen®, UK). The resulting gDNA was used in the qrtPCR assay. The qrtPCR were performed using a BI PRISM 7500 Sequence Detection System (Applied BioSystems, UK). The total volume of reactions was 20 μl containing 2 μl gDNA, 10 μl of 2x SYBR® premix. The following universal 16S bacterial primers were used 357f CTCCTACGGGAGGCAGCAG and 519r GAATTACCGCGGCTGCTG to amplify 16S bacterial rRNA gene [35]. The S7 gene served as an internal standard (AgS7 forward, GTGCGCGAGTTGGAGAAGA; AgS7 reverse, ATCGGTTTGGGCAGAATGC). Each target was quantified in duplicate and the threshold crossing values (Ct-values) of the samples were first standardized (using a standard curve) and were then normalized to the geometric mean of mosquito.

#### Gene silencing with RNAi technique

We silenced two type III fibronectin genes, *FN3D1* (*AGAP005147*) and *FN3D3* (*AGAP001824*), and the gustatory receptor gene *GR9*, (*AGAP009805*). To synthesize double stranded RNA (dsRNA), a fragment of target gene was PCR cloned using gene specific T7 primers from a cDNA library that was synthesized from tRNA extracted from 10 mosquitoes using TRIzol reagent (Life Technologies, UK). The dsRNAs production was performed using the MEGAscript T7 Kit (Ambion, UK). Purified dsRNAs (using RNeasy kit, QIAgen®, UK) was concentrated to a 3 μg/μl and a 69 nl dsRNA was injected into the lateral side of the thorax of female mosquitoes [36]. The control mosquitoes were injected with dsRNA of the *LacZ* gene (*dsLacZ*).

#### Statistical analysis

The bacterial count data were highly skewed, thus counts were log transformed before statistical analysis. Statistical analysis was performed using a linear fixed effects model. For bacterial proliferation data, the bacterial count was considered as a function of time after blood meal, with time as a categorical fixed effect. For gene knockdown effects, the bacterial count was considered as a function of gene knock down with gene as a categorical fixed effect. The repeatability of the FCM and qrtPCR methods is summarized by the coefficient of variation, which is used regularly as a measure of precision or assay variability [37].

## Results and discussion

### Optimization of the staining protocol

**Fig. 1:**
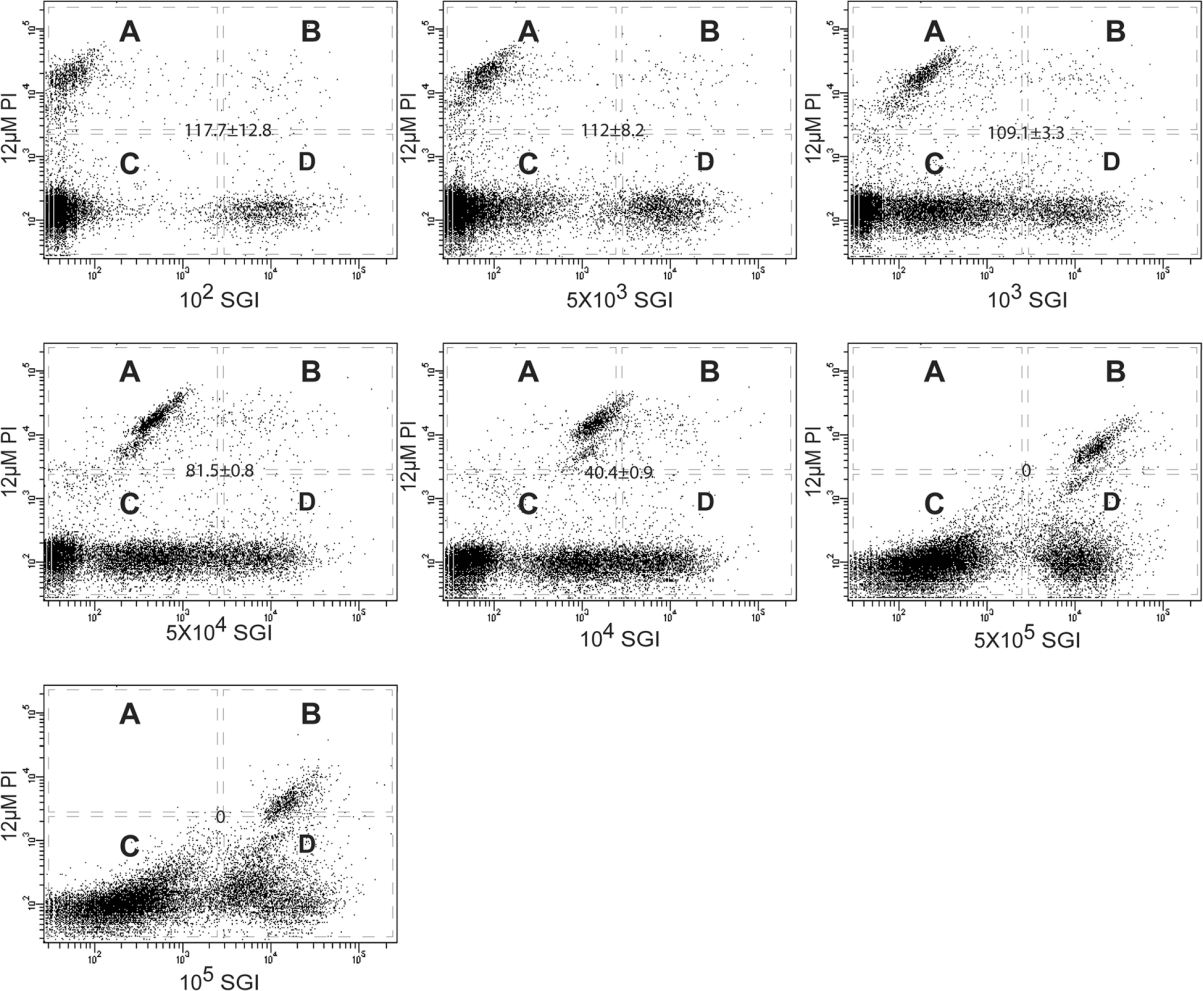
SYBR Green I (SGI) serial dilution to determine optimum dilution rate for discrimination between cells and debris and between live and dead microbiota in the mosquito midgut. Each scattergram represent flow cytometric dot plots of red (FL3) versus green (FL1) fluorescence of midgut homogenate suspension stained concurrently with different SGI dilution with respect to a fixed PI at fixed concentration (12 μM) as recommended by the manufacturer. Four regions in the scattergram (A, B, C, D) represent different population of FCM collection, i.e. (A) PI intensive cells representing dead cells, (B) both PI and SGI positive cells representing bacterial cells with partially compromised membrane, (C) background noise and autofluorescing debris and (D) SGI intensive cells representing live bacteria. The number at the center of the plot correspond to the ratio of mean fluorescent intensity (±95 % CI) between bright and dim bacterial cell populations on the SYBR channel

Dual SGI (green) and PI (red) staining results in intense green fluorescence and dim red fluorescence of live bacterial cells, and intense red and dim green fluorescence of dead bacterial cells. Due to a high concentration of debris in midgut homogenates, particularly heme (the most abundant debris), autofluorescence can limit the signal resolution for the SGI as well as the PI channels depending up on the dye concentration. To address this, we optimized the concentrations of the fluorochromes. First, a range of SGI dilutions (10^2^, 5 × 10^3^, 10^3^, 5 × 10^4^, 10^4^, 5 × 10^5^, and 10^5^) were tested in combination with a fixed PI concentration (12 μM) in mosquito midgut homogenates spiked with live and killed bacterial cells. The FCM collections were displayed on a bivariate dot plot with the SYBR emission on the abscissa and the PI emission on the ordinate axis (Fig. 1).

The ratio of SGI median fluorescence intensities (MFI) of bright to dim bacterial population to quantify the capacity to discriminate between live and dead cells (MFI expressed as median value in relative fluorescence units). Our data suggests that the MFI for GSI increased with the dye concentration until the fluorescence signal was saturated, indicating a high binding affinity of this fluorochrome to nucleic acids [37]. There was a clear separation between bacterial cells and debris at the SGI dilution of 10^2^ and 5 × 10^3^. The most optimum discrimination was achieved at the 5 × 10^3^ dilution, indicating a complete FRET from SGI to PI [33]. This SGI concentration is much higher than the recommended, i.e. 10^4^.

**Fig. 2:**
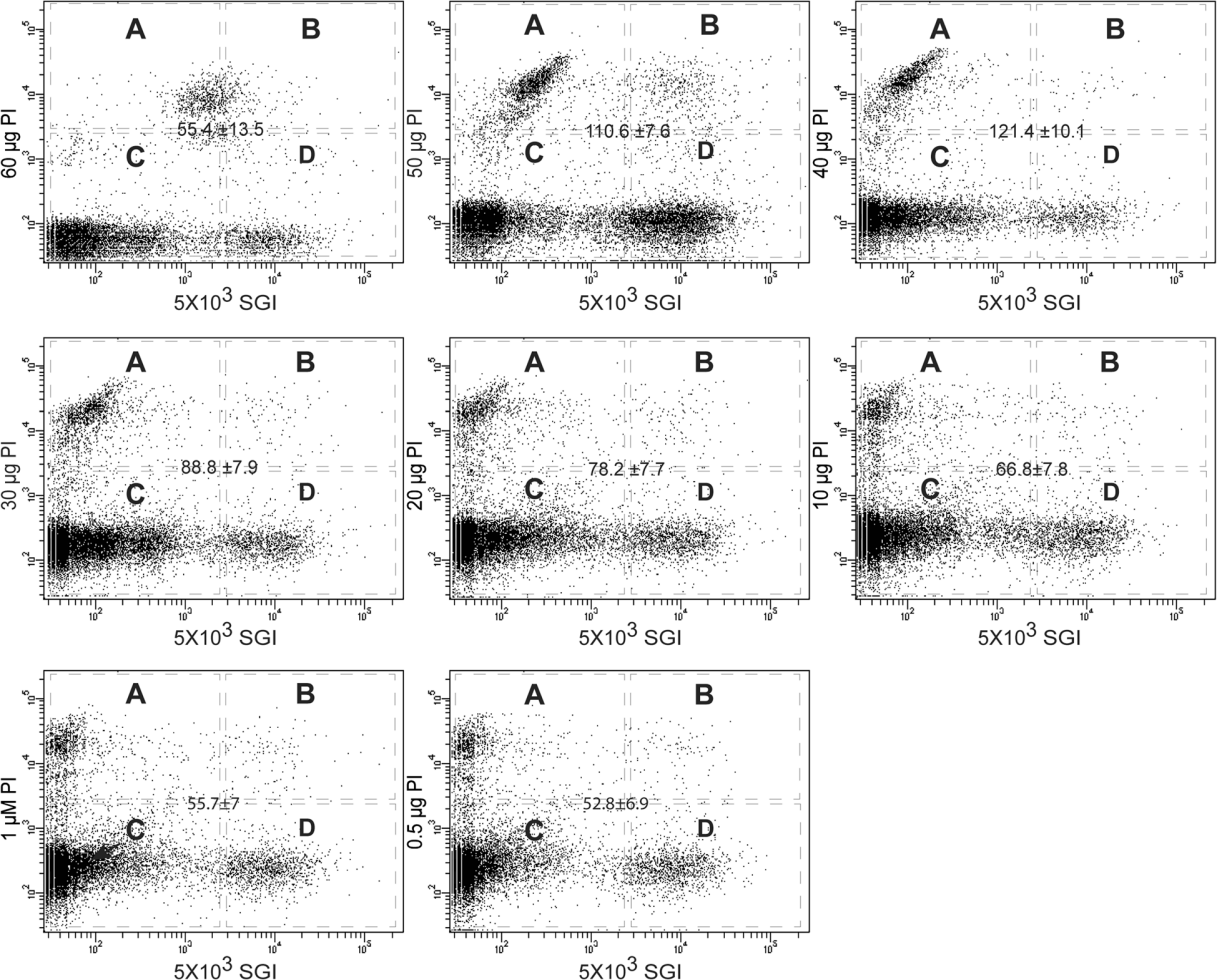
Propidium Iodide (PI) serial dilution to determine optimum dilution rate for discrimination between cells and debris and between live and dead microbiota in the mosquito midgut. Each scattergram represent flow cytometric dot plots of red (FL3) versus green (FL1) fluorescence of midgut homogenate suspension stained concurrently with different PI dilutions with respect to a fixed SGI concentration (5 × 10^3^). Four regions in the scattergram (A, B, C, D) represent different population of FCM collection, i.e. (A) PI intensive cells representing dead cells, (B) both PI and SGI positive cells representing bacterial cells with partially compromised membrane, (C) background noise and autofluorescing debris and (D) SGI intensive cells representing live bacteria. The number at the center of the plot correspond to the ratio of mean fluorescent intensity (MFI) (± 95 % CI) between bright and dim cells populations on the PI channel

Next, we tested a range of PI concentrations (60 μM, 50 μM, 40 μM, 30 μM, 20 μM, 10 μM, 1 μM, and 0.5 μM) with respect to a fixed SGI concentration (5 × 10^3^) in order to identify the optimum PI concentration for clear discrimination between cells and debris and also between live and dead cells. The FCM collections are reported in Fig. 2. The results showed a clear differentiation between cells and debris for all PI dilutions, except for 60 μM, where PI positive cells were poorly detected perhaps due to increased background fluorescence [38]. A decreased MFI observed at higher PI concentrations might be related to a leakage of the dye from cells leading to increased background fluorescence [39].

From the two optimization tests, we identified SGI 5 × 10^3^ and PI 30 μM as the optimum dye combination for subsequent FCM analysis.

### Live dead discrimination

**Fig. 3:**
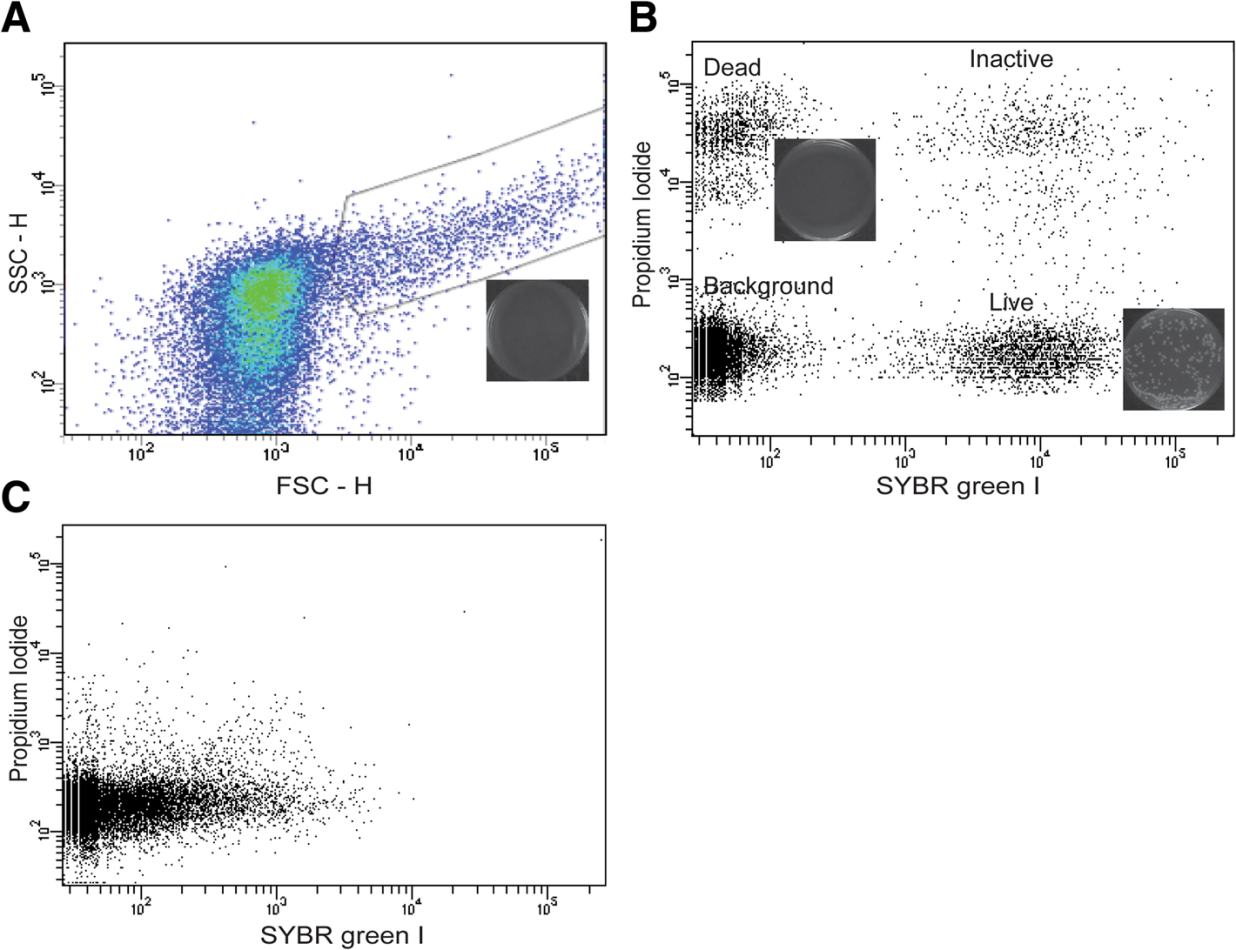
Flow cytometric analysis of midgut homogenate in the blood fed mosquito. **a** Total FCM collection depicted in SSC *vs* FSC plot, showing a large population of events with low FSC and SSC, and a smaller population accounting for 3–4 % with higher FSC and SSC. **b** SYBR *vs* PI dot plot of low FSC and SSC population, showing four distinct populations depending up on their fluorescein characteristics. **c** SYBR *vs* IP dot plot of low FSC and SSC population in aseptic mosquito treated with a cocktail of antibiotics, showing depletion of all the bacterial cells. Insets represents LB agar plate seeded with FCM sorts from the corresponding population

After dual SGI/PI staining, midgut homogenates were analysed by FCM and the resulting collections were first displayed on scattergram, i.e. forward scatter (FSC) *vs* side scatter (SSC). The plot revealed two distinct populations: a large population of events with low FSC and SSC, and a smaller population accounting for 3–4 % of the total with higher FSC and SSC (Fig. 3a). Microscopic examination of the sorts of the latter population revealed cells with relatively large nuclei, probably representing midgut epithelial cells, and also aggregates of debris from digested erythrocytes [40, 41]. There was no colony growth when sorts from this population were seeded on solid agar media (inset in Fig. 3a). These events were gated out from subsequent analyses.

The populations exhibiting low FSC and SSC were analysed using a bivariate dot plot displaying the SYBR emission on the abscissa and the PI emission on the ordinate axis (Fig. 3b). This analysis reproducibly produced four discrete populations, including a non-fluorescent population (left-bottom corner), and three fluorescent populations. Visualization of sorts of the non-fluorescence population under a light microscope revealed that no bacteria cells were present and no bacterial colony growth was observed from sort of this population when seeded on solid agar media. The three fluorescent populations fall in separate regions in the fluorescence scattergram: (1) Bottom-right corner: live SYBR positive cells that when streaked on LB agar plates resulted in growth of bacterial colonies (inset in Fig. 3b). These bacterial colonies demonstrate a great potential of combining FCM with other microbiological techniques for detailed characterization of the microbiota community. (2) Top-left corner: dead cells only fluorescing on the PI channel. The absence of reproductive growth and metabolic activity relates to a loss of membrane integrity in bacterial cells [42]. While both SGI and PI can enter dead cells due to compromised outer membrane, these cells fluorescence only red in the presence of saturating PI concentrations. This is due to FRET whereby the fluorescent emission spectrum of SYBR is absorbed by PI and no longer visible [33]. As expected, no colony growth on the plates seeded with the PI positive sorts (see inset). (3) Top-right corner: cells that are potentially partially permeable to PI, hence fluoresce both SYBR and PI because of incomplete FRET from SGI to PI [33]. A rapid cell division and cell-wall synthesis during the exponential growth phase can create transient perforations of the cell-wall mediating entry of PI [43]. Sort-collection from this population was streaked on LB agar plates, but no colony was observed, which might be due to PI toxicity of the cells. To further demonstrate that the three fluorescence populations constitute bacterial cells, FCM analysis was conducted on midgut homogenates isolated from aseptic mosquitoes. The FCM collection is depicted in Fig. 3c, which shows the depletion of the three fluorescence populations. This further confirmation that the double positive population correspond to bacterial cells.

### Validation of FCM to measure midgut bacteria load

**Fig. 4:**
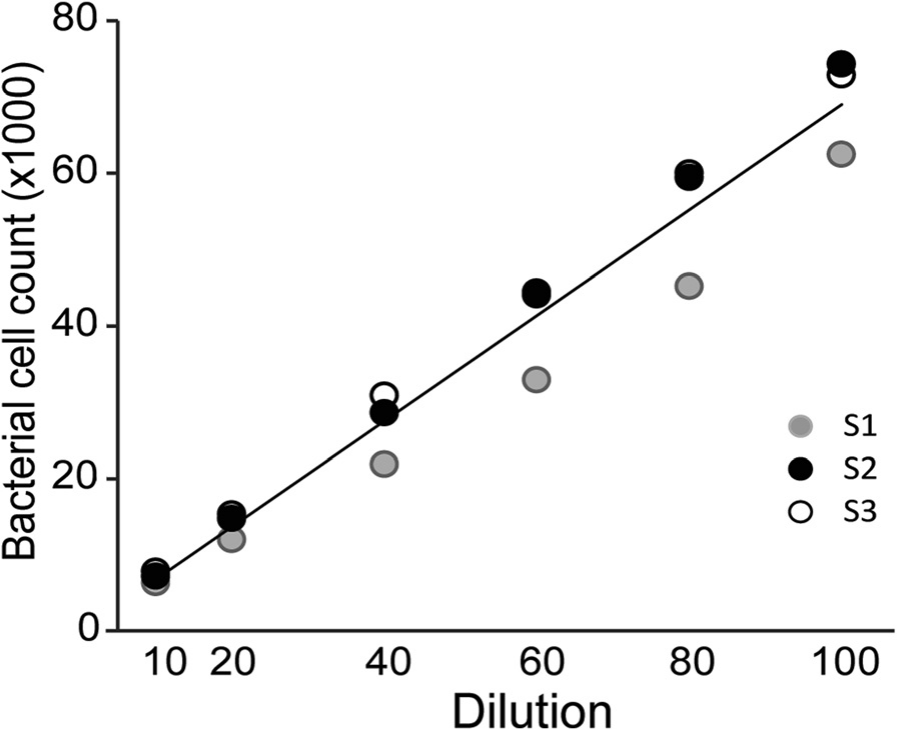
Validation of FCM to quantify bacterial in midguts of mosquito. Regression plot depicting serial dilution of gut homogenate *vs* bacterial count to show the linearity of flow cytometry measurement (*R* ^2^ = 0.987). The test was repeated thrice

We determined the linearity of the FCM measurement of microbiota on a serial dilution of midgut homogenates. The number of microbiota as a function of the dilution factor is presented in Fig. 4. A strong linear relationship of the counts in the serial dilutions and the dilution factor was observed (*R*^2^ = 0.987). The FCM measurement of gut microbiota has a better precision compared to qrtPCR, with co-efficient of variation (CV) 0.56 and 1.36, respectively, for the two measurements. Taken together, these results confirm that FCM is an accurate analytical tool for applications in mosquito midgut microbiology.

### FCM measurement of bacterial load in blood fed mosquito

The determination of the abundance of the microbial 16S rRNA gene using qrtPCR has been routinely applied in mosquito midgut microbiology including determination of the effect of mosquito gene silencing on microbiota proliferation [13] or determination of the microbiota proliferation and dynamics during the mosquito gonotropic cycle [44]. We assessed the capacity of the FCM technique to derive such measurements.

**Fig. 5:**
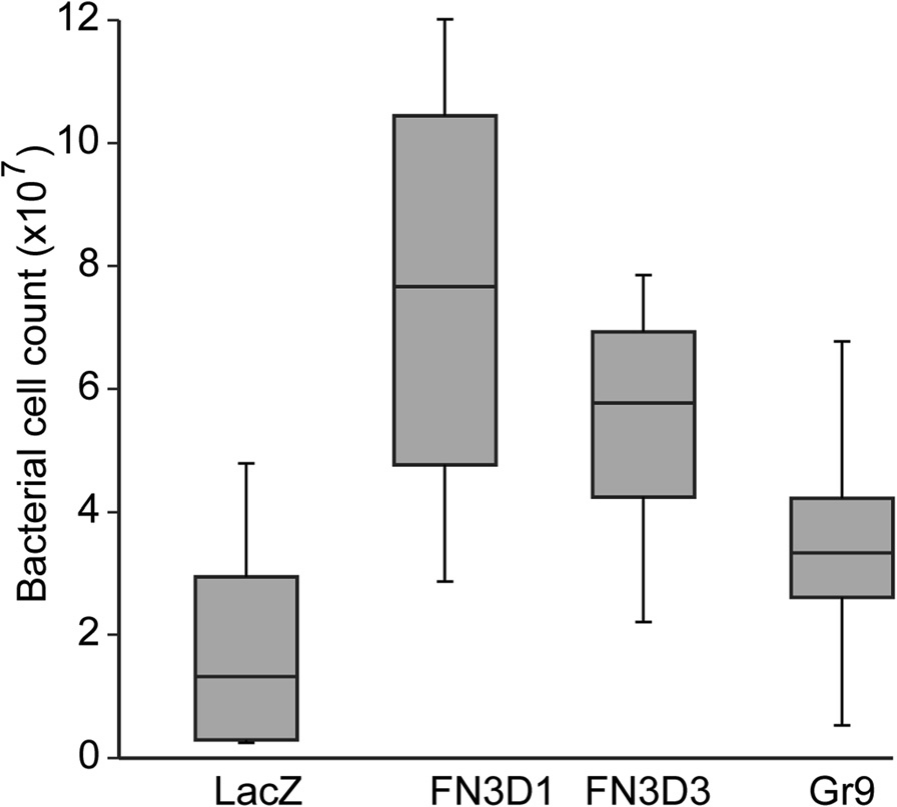
Box plot depicts median number of bacterial with first and third quartiles. Samples correspond midgut homogenates from epithelial receptor gene silenced mosquito

We silenced the same set of genes as in [13], i.e. *GR9*, *FN3D1* and *FN3D3*, in *An. gambiae* mosquitoes and enumerated the microbiota 24 h after blood feed. The results showed that the number of microbiota was significantly higher in silenced than control (*dsLacZ*-injected) mosquitoes for all the genes tested (*P* < 0.001; Fig. 5). The FCM technique reproduces the previous qrtPCR-based results.

**Fig. 6:**
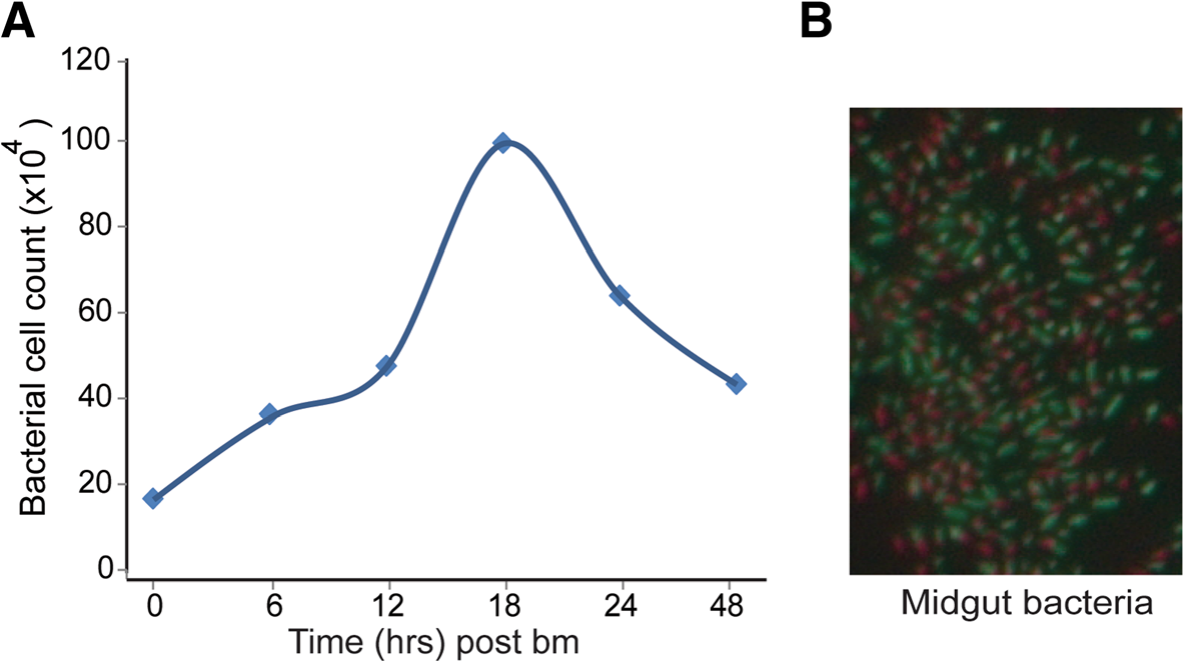
FCM quantification of bacterial in midguts of blood fed mosquito. **a** The dynamics of midgut bacterial over gonotropic cycle; **b** Depicting live (green) and dead (red) bacteria in the midgut lumen

We also used the FCM technique to determine the proliferation of mosquito midgut microbiota at different time points after blood feeding. The result showed that the microbiota reached a peak load at 18 h post-feeding and started declining at 24 h (Fig. 6a). This peak load is significantly earlier than what is reported in a previous study, where microbiota appear to peak at 30 h [44]. To elucidate whether differences are due to limitations of the qrtPCR technique, we firstly carried out direct microscopic examination of midgut smears stained with SYBR and PI. The results revealed that at 24 h post-feeding a significant proportion of the microbiota cells were non-viable albeit still intact (Fig. 6b). Such dead cells can contribute to the qrtPCR quantification, while once they start lyse they release free microbial nucleic acids that deposit and build up in the gut lumen [45–47]. We examined the latter by measuring the abundance of extracellular 16S rRNA gene in the midgut using qrtPCR and found that free 16S gene accounts for up to 9 % of cell counts (data not showed).

### FACS can enumerate microbiota in fixed midgut samples

**Fig. 7:**
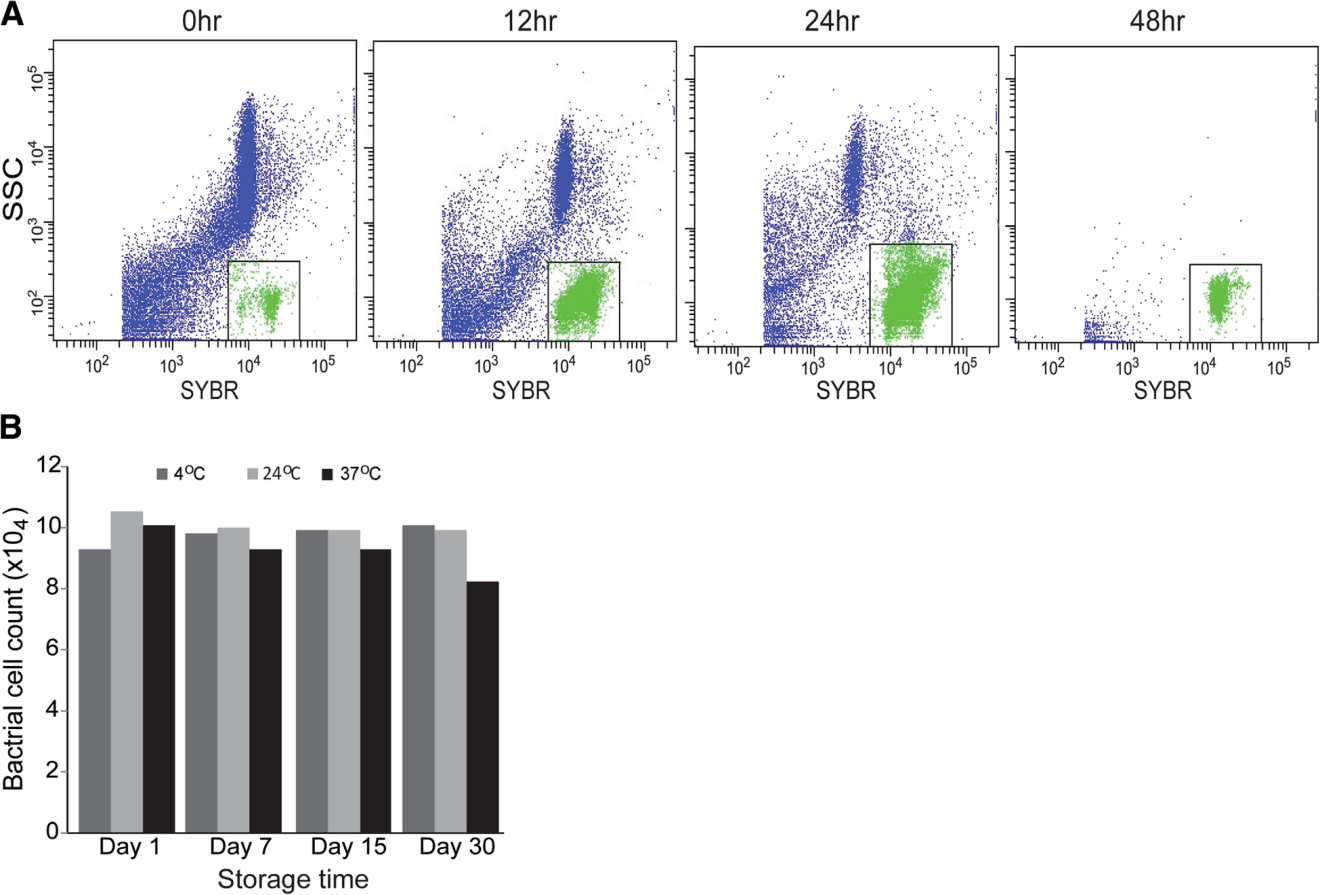
Effect of fixation of midgut samples on the FCM microbiota analysis. **a** Dot plot of SSC *vs* SYB of Flow cytometry collection from fixed midgut homogenate at different time points after blood feed. The bacterial event population is shown in box. **b** Effect of storage conditions of fixed gut homogenate samples on the flow cytometry bacterial count. The samples correspond to midgut homogenates fixed with 4 % PFA in PBS

We explored the possibility of applying FCM to enumerate the microbiota in fixed midgut samples that were homogenized in 4 % PFA and passed through the FCM after staining with SGI (5 × 10^3^). The results were displayed on SSC *vs* SYBR dot plots (Fig. 7a), and revealed that bacterial cells express a strong SYBR fluorescence and low side scatter. Populations with high SSC and considerable SYBR fluorescence were sorted and confirmed microscopically to be debris. The high fluorescence in this population is attributable autofluorescence due to heme aggregates [48]. This population reduced drastically with the progression of blood meal digestion. Often mosquito samples are collected from field sites, fixed and transported to the laboratory for analysis; therefore a significant time lapse passes between collection and analysis of samples. We compared the FCM recovery rates of bacteria in fixed midguts stored at three different temperatures for three different time periods. The measurement at day 0 was considered as baseline number. No significant differences were detected for samples stored at 4 °C, 24 °C or 37 °C, for 1, 7, 15 or 30 days post-fixation (Fig. 7b). These data demonstrate that the efficiency of the FCM technique to measure total microbiota cells is not affected by fixation and subsequent storage of the samples for prolonged time periods.

## Conclusion

Here we have demonstrated that FCM analysis in conjunction with SGI/PI dual staining can robustly discriminate between live and dead microbiota cells, and can separate both types of cells from autofluorescing debris, in a highly reliable and precise fashion. The technique can be applied directly on midgut homogenates, and thus offers a rapid and inexpensive option compared to qrtPCR in quantifying the microbiota as it bypasses intermediate steps such as extraction of DNA or RNA and conversion to cDNA in the latter case. Depending on the availability of species-specific antibody, however, the FCM technique can be used for differential detection and quantification of gut microbiota to the species level can be achieved. In clinical microbiology samples, FCM combined with antibody labelling has been used to sort live recombinant mycobacterial mutants with high expression of foreign inserts and to enrich those sorted bacterial populations [49]. It can be also combined with other state-of-the-art microbiological techniques for the molecular taxonomic identification of specific bacterial populations and potentially their spatial distribution, temporal dynamics, and physiology [50–53] or to study the effect of a procedure such as gene silencing or mosquito blood meal and infection.

## Abbreviations

CV: Co-efficient of variation
DNA: Deoxyribonucleic acid
FCM: Flow cytometry
FN3D1: Fibronectin type III domain- protein 1
FN3D3: Fibronectin type III domain- protein 3
FRET: Fluorescence resonance energy transfer
FSC: Forward Scatter
GR9: G protein coupled receptor protein 9
MFI: Mean fluorescence intensity
PBS: Phosphate-buffered saline
PCR: Polymerase chain reaction
PFA: Paraformaldehyde
PI: Propidium Iodide
qrtPCR: Quantitative real-time PCR
RNA: Ribonucleic acid
SGI: SYBR green I
SSC: Side Scatter
